# Sonic Hedgehog Signaling in Limb Development

**DOI:** 10.3389/fcell.2017.00014

**Published:** 2017-02-28

**Authors:** Cheryll Tickle, Matthew Towers

**Affiliations:** ^1^Department of Biology and Biochemistry, University of Bath Bath, UK; ^2^Department of Biomedical Science, The Bateson Centre, University of Sheffield Western Bank, Sheffield, UK

**Keywords:** Sonic hedgehog, limb, digits, mouse, chick, positional information

## Abstract

The gene encoding the secreted protein Sonic hedgehog (Shh) is expressed in the polarizing region (or zone of polarizing activity), a small group of mesenchyme cells at the posterior margin of the vertebrate limb bud. Detailed analyses have revealed that Shh has the properties of the long sought after polarizing region morphogen that specifies positional values across the antero-posterior axis (e.g., thumb to little finger axis) of the limb. Shh has also been shown to control the width of the limb bud by stimulating mesenchyme cell proliferation and by regulating the antero-posterior length of the apical ectodermal ridge, the signaling region required for limb bud outgrowth and the laying down of structures along the proximo-distal axis (e.g., shoulder to digits axis) of the limb. It has been shown that Shh signaling can specify antero-posterior positional values in limb buds in both a concentration- (paracrine) and time-dependent (autocrine) fashion. Currently there are several models for how Shh specifies positional values over time in the limb buds of chick and mouse embryos and how this is integrated with growth. Extensive work has elucidated downstream transcriptional targets of Shh signaling. Nevertheless, it remains unclear how antero-posterior positional values are encoded and then interpreted to give the particular structure appropriate to that position, for example, the type of digit. A distant cis-regulatory enhancer controls limb-bud-specific expression of *Shh* and the discovery of increasing numbers of interacting transcription factors indicate complex spatiotemporal regulation. Altered Shh signaling is implicated in clinical conditions with congenital limb defects and in the evolution of the morphological diversity of vertebrate limbs.

## Introduction

Over 20 years ago the first evidence was presented that *Sonic hedgehog* (*Shh*), an orthologue of the *Drosophila Hedgehog* (*Hh*) gene, encodes the long sought after morphogen that specifies antero-posterior pattern in developing vertebrate limbs (Riddle et al., 1993). Grafting experiments in chick wing buds in the 1960s revealed that a group of morphologically indistinguishable mesenchyme cells at the posterior margin of the wing bud (the margin nearest the tail), later known as the polarizing region (or zone of polarizing activity), is an important cell-cell signaling center that controls development across the antero-posterior axis (Saunders and Gasseling, 1968). Tissue transplanted from the posterior margin of one chick wing bud to the anterior margin of another was shown to have the striking ability to duplicate the pattern of three digits, so that another set develop in mirror-image symmetry to the normal set. Based on these observations it was proposed that the polarizing region produces a diffusible morphogen that specifies antero-posterior positional values (Wolpert, 1969). These positional values are interpreted so that a structure, such as a digit with an appropriate identity, develops in the correct position.

**Figure 1:**
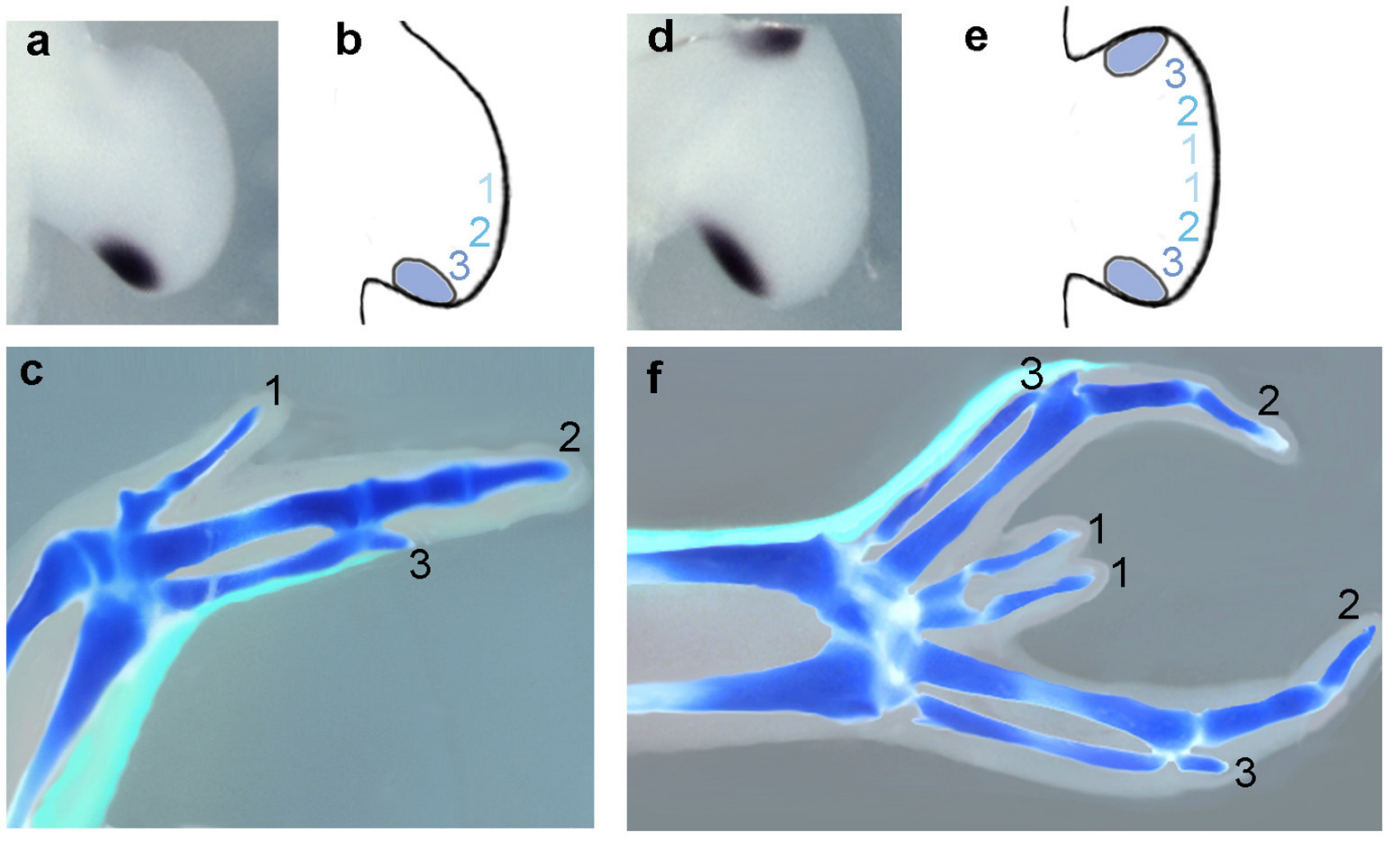
**Shh as a morphogen in the chick wing bud. (a)** *Sonic hedgehog* (*Shh*) expression in the polarizing region at the posterior margin of the early chick wing bud (Riddle et al., 1993). **(b)** A gradient of Shh in the chick wing bud (blue shaded numbers) specifies antero-posterior positional values for three digits (1, 2, and 3) in cells adjacent to polarizing region over 12 h. **(c)** Chick wing digit skeleton with polarizing region descendants fate-mapped by GFP-expression (green) (Towers et al., 2011). Digits form in tissue adjacent to descendants of the polarizing region that form narrow strip of cells along posterior wing margin. **(d)** Chick wing bud with anterior polarizing region graft expresses *Shh* at both anterior and posterior margins (Towers et al., 2011). **(e)** Mirror-image symmetrical positional values specified as in **(b)** as a result of Shh being produced by both graft and host. **(f)** Chick wing digit skeleton pattern with grafted polarizing region **(d)** and progeny fate mapped by GFP expression (Towers et al., 2011). Six digits form in an anterior to posterior pattern 3-2-1-1-2-3 and grafted polarizing region descendants form narrow strip of cells along anterior wing margin. In all cases, data shown is representative of data in the original cited papers.

The key pieces of evidence that Shh is the polarizing morphogen are that *Shh* transcripts were found to be localized to the polarizing region of the chick wing bud (Figures 1a–f) and that *Shh*-expressing cells grafted to the anterior margin of chick wing buds can produce the same effects as grafts of the polarizing region (Riddle et al., 1993). Earlier experiments revealed that tissue from the posterior margin of mammalian limb buds grafted to the anterior margin of chick wing buds could duplicate the pattern of chick wing digits (Tickle et al., 1976; Fallon and Crosby, 1977). This is explained by the finding that *Shh* is expressed at the posterior margin of mammalian limb buds (Echelard et al., 1993; Odent et al., 1999). *Shh* has now been shown to be expressed at the posterior margin of the limb buds of all vertebrates studied to date, including the fin buds of the most primitive chondrichthyan fishes such as the shark (Dahn et al., 2007).

Experiments in which the polarizing region was grafted to the anterior margin of another chick wing bud showed that polarizing region signaling also plays a role in controlling the width of the limb bud and that widening of the bud is required to specify a complete set of new antero-posterior positional values (Tickle et al., 1975; Smith and Wolpert, 1981). The earliest detected effect of a polarizing region graft was an increase in cell proliferation in adjacent mesenchyme in the host wing bud (Cooke and Summerbell, 1980). In addition, it was proposed that the polarizing region controls the production of a factor by the mesenchyme that maintains the apical ectodermal ridge over the region of the wing bud that will give rise to distal structures including the digits (Zwilling and Hansborough, 1956). The apical ectodermal ridge is a signaling region that rims the bud and is required for proximal-distal patterning and outgrowth and the laying down of structures along this axis; the extent of the apical ectodermal ridge across the antero-posterior axis controls the width of the wing bud and determines the number of digits that can form. The effects of the polarizing region on the apical ectodermal ridge also link antero-posterior and proximo-distal pattern formation. This explains the observation that polarizing region grafts made at later stages of development affect the antero-posterior pattern of more-distal structures (Summerbell, 1974).

Early experiments highlighted the complex relationship between the polarizing region and apical ectodermal ridge. In order for a polarizing region to signal, it has to contact the apical ectodermal ridge (Tickle et al., 1975) and this interaction is required in order for the polarizing region to maintain production of the apical ridge maintenance factor by the mesenchyme that will form distal structures. In addition, in the chick wing bud, the polarizing region itself demarcates the posterior limit of the apical ectodermal ridge and grafts of the polarizing region placed under the apical ectodermal ridge flatten it (Saunders and Gasseling, 1968). Interestingly, it has also been shown that the dorsal ectoderm of the wing bud, which produces a signal controlling the development of the dorsal pattern of structures (e.g., extensor muscles), is also required for the polarizing region to signal (Yang and Niswander, 1995). Thus, signaling along all three axes of the developing limb bud is integrated.

It has now been shown that Shh affects cell proliferation in the chick wing bud by controlling expression of genes encoding cell cycle regulators including D cyclins independently of the apical ectodermal ridge (Towers et al., 2008). Work on mouse limb development has shown that Shh controls expression of the *Gremlin1* gene, which encodes the BMP antagonist that acts as the apical ridge maintenance factor (Zuniga et al., 1999). In addition, it has also been demonstrated that short-range Shh signaling can flatten the apical ridge above the polarizing region (Bouldin et al., 2010).

Experiments on chick wing buds have identified FGFs as the apical ectodermal ridge signals that promote outgrowth and also maintain *Shh* expression in the polarizing region (Laufer et al., 1994; Niswander et al., 1994). Genetic experiments in mouse have identified Wnt7a as the dorsalizing signal that also contributes to regulating *Shh* expression (Parr and McMahon, 1995). Loss of Wnt7a function in the mouse limb results in the transformation of dorsal to ventral fates and loss of posterior digits (Parr and McMahon, 1995). This second phenotype is consistent with a function for Wnt7a in controlling *Shh* expression since no digits form in the fore-limbs of *Shh*^−/−^ mouse embryos and only a single digit—considered to be an anterior digit 1—is present in hind-limbs (Chiang et al., 1996).

In this review, we will emphasize the parallel contributions that experimental chick embryology and mouse genetics have played in providing the current picture of Shh function in the limb. We will provide an in-depth picture of how Shh specifies antero-posterior positional values in the limb buds of these two main vertebrate models and how this is integrated with its role in growth. We will consider how *Shh* expression in the limb is initiated, maintained and eventually extinguished and how cells respond to the Shh signal. We will finally review clinical conditions affecting the limb and examples of evolutionary diversification of limb morphology that are associated with changes in Shh signaling.

## Specification of antero-posterior pattern

### Chick wing

Detailed embryological experiments on the chick wing bud have been crucial in establishing the signaling parameters of the polarizing region morphogen. The polarizing region was first discovered in the chick wing bud, where it overlaps with a region of programmed cell death, known as the posterior necrotic zone (Saunders and Gasseling, 1962). Indeed, the original grafting experiments were designed to investigate how this region of cell death is controlled (Saunders and Gasseling, 1968). Tissue from the posterior margin of a chick wing bud was grafted to the anterior margin of a second wing bud and this resulted in a mirror-image pattern of digits across the antero-posterior axis. The normal chick wing has three digits (designated at this time as 2, 3, and 4) but following a polarizing region graft to the anterior margin, six digits can develop in the pattern 4-3-2-2-3-4. Note that recent evidence supports numbering of the digits as 1, 2, and 3 (Towers et al., 2011), and this numbering system is now generally accepted and will be used in this review. This grafting experiment provided an assay for polarizing activity and antero-posterior pattern that could readily be scored by the distinct skeletal morphology of each of the three digits of the chick wing. It should be noted that grafts of the polarizing region also affect the antero-posterior pattern of the wing fore-arm skeleton and soft tissues (Shellswell and Wolpert, 1977; Robson et al., 1994). Thus, following a polarizing region graft, two ulnae develop and the pattern of muscles is also duplicated. The myogenic cells of the muscle originate in the somites and migrate into the limb bud but the pattern of the wing muscles is dictated by the connective tissue, which is derived from the lateral plate mesoderm (Chevalier and Mauger, 1977). Therefore, the duplicated pattern of muscles following a polarizing region graft will be based on the response of the cells that give rise to the muscle connective tissue.

The experimental parameters determined for polarizing region signaling in the chick wing (reviewed in Towers and Tickle, 2009) are consistent with the suggestion that the polarizing region produces a long-range morphogen that sets up a concentration gradient across the antero-posterior axis of the wing bud and specifies positional values (Wolpert, 1969). According to this model, the positional values at particular threshold concentrations govern digit identity, with the highest threshold concentration in tissue closest to the polarizing region specifying the most-posterior digit, digit 3, and the lowest threshold concentration in tissue further away specifying the most-anterior digit, digit 1. Thus, any candidate molecule for the polarizing region morphogen must act in a concentration-dependent manner (Tickle, 1981) and provide a long-range signal (Honig, 1981).

The first defined molecule found to mimic the duplicating activity of polarizing region grafts was the vitamin A derivative, retinoic acid (Tickle et al., 1982, 1985) but it was subsequently shown that retinoic acid acts indirectly (Noji et al., 1991; Wanek et al., 1991) by inducing *Shh* expression (Riddle et al., 1993). There is now good evidence that Shh acts in a concentration-dependent fashion to induce digit duplications. When *Shh*–expressing cells, or beads soaked in bacterially produced ShhN protein (the active N-terminal fragment produced by autocatalytic cleavage of the large precursor Shh protein), are placed at the anterior margin of a chick wing bud, the extent of digit duplication depends on the number of *Shh*–expressing cells grafted or the concentration of ShhN protein in which the beads are soaked (Yang et al., 1997). Fewer *Shh*-expressing cells or lower concentrations of Shh elicit duplication of only the anterior digit 1 (Yang et al., 1997). Grafts of *Shh*-expressing cells that induce full digit duplications were also shown to result in two ulnae developing in the forearm together with a duplicated pattern of muscles (Duprez et al., 1999).

The original model for how antero-posterior values are specified in the chick wing bud did not consider the dynamic nature of the process, although experiments showed that the extent of duplication following a polarizing graft depended on the length of time that the graft was left in place (Smith, 1980). A similar time dependency was subsequently seen with Shh–soaked beads (Yang et al., 1997). Furthermore, fate mapping experiments showed that cells near a Shh-soaked-bead give rise to an anterior digit 1 when the bead is removed after a short time, but give rise to a more posterior digit (2) if the bead is left in place for longer (Yang et al., 1997). This process by which positional values of cells change over time in response to an increasing concentration of morphogen is known as promotion (see also (Gurdon et al., 1995). An alternative process in which wing bud cells acquire a stable positional value depending on the duration of Shh signaling and then are displaced by growth can be ruled out because an anterior digit 1 has been shown to arise in tissue which was not originally adjacent to a polarizing region graft (see Tickle, 1995).

The parameters of polarizing region discussed above were determined in experiments in which additional digits were induced following polarizing region grafts to the anterior margin. But what is the evidence that Shh acts long range and how does Shh signaling specify antero-posterior positional values during normal development of the chick wing? Measurements of Shh activity in slices taken from different positions across the bud using an *in vitro* cell-differentiation assay are consistent with there being a concentration gradient of Shh across the bud, with Shh activity of a posterior slice being 5–6 times higher than that of a middle slice (Zeng et al., 2001). Another indication that Shh spreads across the wing bud and provides a long range signal is that high levels of the transcripts of known direct gene targets of Shh signaling, including *Ptch1* (encoding the main receptor for Shh), and *Gli1* (encoding a transcriptional effector of Shh signaling) encompass the posterior two-thirds of the wing bud, including adjacent tissue in addition to the polarizing region (Marigo et al., 1996). It should also be noted that following a polarizing region graft or implantation of an Shh bead to the anterior margin of the chick wing, there is a burst of high level *Ptch1* expression in the anterior part of the wing bud, which then subsides and is later followed by the establishment of a stable domain of high level *Ptch1* expression (Drossopoulou et al., 2000). This suggests that cells could respond to and interpret two waves of Shh signaling; the first defining the size of the domain that can give rise to digits, and the second, promoting the growth of this domain and specifying positional values.

**Figure 2:**
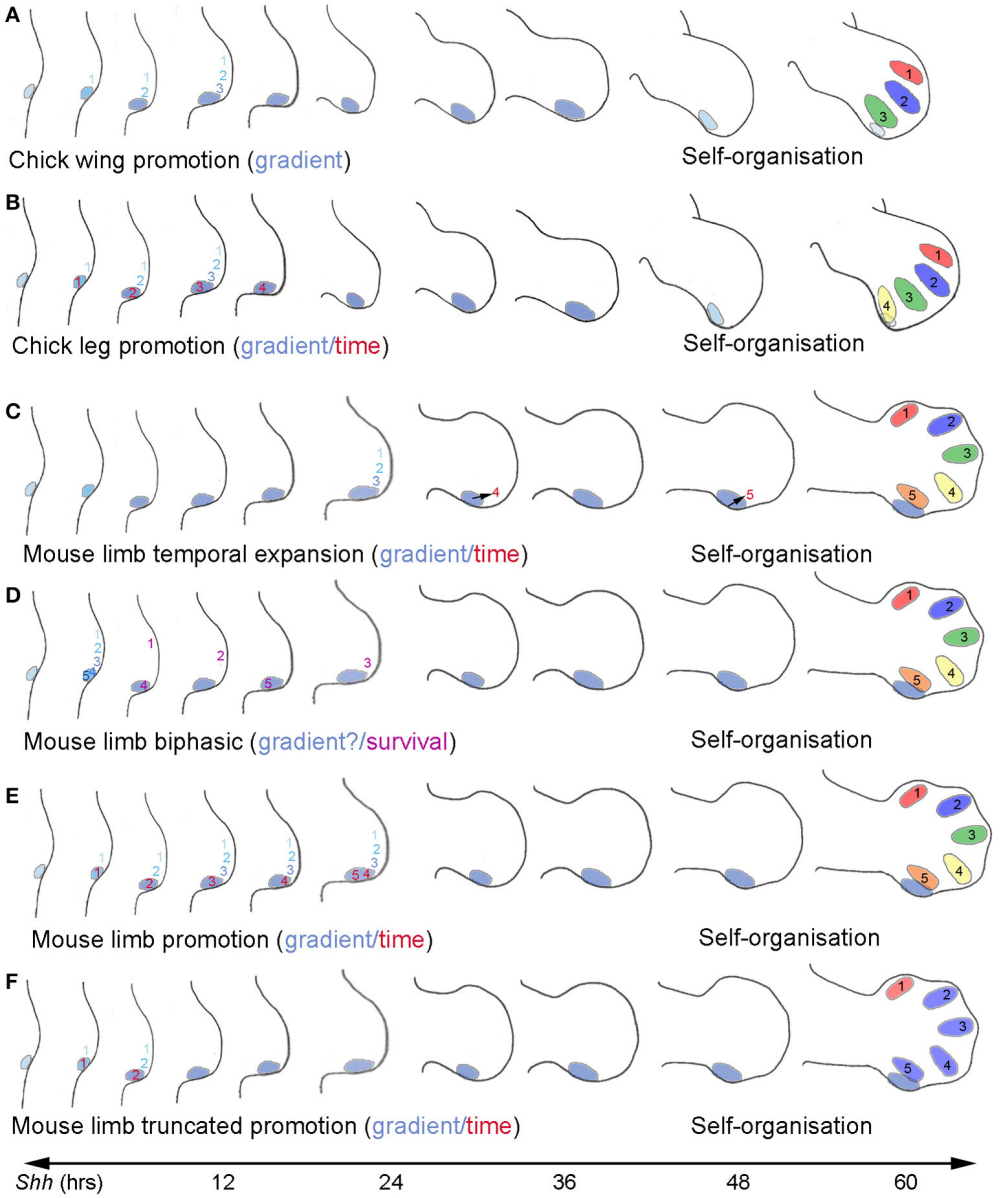
**Comparison of models of Shh function in chick and mouse limbs. (A)** Chick wing promotion model. Positional values of digits 1, 2, and 3 specified adjacent to polarizing region (blue shading) and promoted over 12 h through a series of increasingly posterior positional values by a concentration gradient of paracrine Shh signaling (graded blue shading—note coloring of polarizing region also shows strength of *Shh* expression. *Shh* terminated at around 60 h as digit condensations form by self-organization (black numbers). Colors of developing digits indicate a different positional value that cells were specified with. **(B)** Chick leg promotion model. Positional values of digits 1, 2, and 3 specified as **(A)** but polarizing region cells promoted through progressively anterior positional values over 16 h in response to time of autocrine Shh signaling (red numbers) and form digit 4. *Shh* terminated at around 60 h. **(C)** Mouse limb temporal expansion. Positional values of digits 1, 2, and 3 specified adjacent to the polarizing region by a gradient of paracrine Shh signaling over approximately 24 h– it is unclear whether promotion is involved (see **A**). Positional values of digits 4 and 5 specified in polarizing region sometime before *Shh* terminates at 60 h according to duration of autocrine Shh signaling. *Shh* terminates at around 60 h. **(D)** Mouse limb biphasic model. Positional values of digits 1, 2, 3, 4, and 5 specified by Shh, possibly by a gradient of paracrine signaling from the polarizing region in approximately 6 h. It is unclear whether promotion is involved and is possible in this time (see **A**), or if Shh levels can reach concentrations predicted required to specify posterior positional values. Shh signaling over the next 16 h required for specified digit progenitor cells to proliferate and form condensations in the order digit 1, 4, 2, 5, and 3 (purple numbers). **(E)** Mouse limb promotion model. Positional values of digits 1, 2, 3, and 4 specified as **(B)** and polarizing region enlarges sufficiently to give rise to digits 4 and 5 by self-organization. Note promotion model does not easily explain digit 5 patterning that requires a shorter exposure to form than digit 3 (see **D**). **(F)** Mouse limb truncated promotion model. Anterior positional values specified (1 and 2) specified by autocrine and paracrine signaling and then cells become refractory to further posterior promotion. Digits form by self-organization: 1, 2, and 3 from cells adjacent to polarizing region, digits 4 and 5 from the polarizing region.

The temporal specification of positional values specified by Shh in normal wing development has been directly addressed by applying cyclopamine, a small molecule inhibitor of Hh signaling at the level of Smoothened to chick embryos, at a series of short time intervals after the onset of *Shh* expression in wing buds (Towers et al., 2011). Smoothened, a member of the G-protein coupled receptor superfamily, is normally activated upon Shh binding to Ptch1, and this triggers of activation of the Gli family of transcription factors (see section on Mechanisms of Shh signaling). Application of cyclopamine about 4 h after the onset of *Shh* expression results in the development of just the anterior digit 1, the anterior and middle digits (1 and 2) develop when cyclopamine is added at 8 h while a complete set of digits (1, 2, and 3) develop when cyclopamine is added at 12 h (Towers et al., 2011). Furthermore, fate mapping experiments show that promotion is occurring with cells next to the polarizing region first being specified to form the anterior digit 1, then being promoted to form the middle digit 2 and finally the posterior digit 3 (Figure 2A).

The effects of Shh signaling on antero-posterior growth must be included in any comprehensive model for specification of antero-posterior pattern in the chick wing. Application of cyclopamine in the experiments described above demonstrated that Shh signaling has effects on both specification of antero-posterior positional values and growth because this treatment not only prevented promotion but also expansion of the region of the wing bud that will give rise to distal structures leading to the development of fewer digits (Towers et al., 2008, 2011). When growth alone is targeted by adding trichostatin A or colchicine, and following over-expression of the cyclin-dependent kinase inhibitor—*p21*^*Cip1*^—at a similar series of time points, fewer digits also develop, but because specification of positional values and promotion by Shh signaling are unaffected, the digits that develop are posterior digits (Towers et al., 2008). These experiments show that specification of antero-posterior positional values in the early chick wing bud is coupled with growth that determines the width of the wing bud.

The cyclopamine experiments also show that antero-posterior values are specified over a relatively short time period during early wing bud development. However, these values will not be interpreted in terms of digit identity until much later in development when the digit condensations develop (Figure 2A). When the *Shh*-expressing region is completely removed from the early wing bud at the time when the positional values that specify two digits are specified, truncated wings develop with posterior structures being preferentially lost (Pagan et al., 1996), showing the crucial importance of Shh signaling in stimulating antero-posterior expansion and maintaining the apical ectodermal ridge. Resulting skeletons bear resemblance to those of the wings of the chicken mutant *Oligozeugodactyly* (*Ozd*) that develop devoid of Shh (Ros et al., 2003). It is unclear why *Shh* continues to be expressed at the posterior margin of the chick wing bud long after the antero-posterior values have been specified (Figure 2A see section Termination of *Shh* expression).

### Chick leg

The chick leg has four morphologically distinct digits (numbered 1, 2, 3, and 4 in antero-posterior sequence). Early grafting experiments demonstrated that chick leg buds also have a polarizing region but it was noted that when the leg polarizing region was grafted to a chick wing bud, a toe frequently developed in the duplicated wings (Summerbell and Tickle, 1977). It has since been demonstrated using grafts from the Green Fluorescent Protein-expressing transgenic chicken to make fate maps of the polarizing region that the chick leg polarizing region gives rise to the most posterior digit 4, whereas in the chick wing all the digits come from tissue anterior to the polarizing region (Towers et al., 2011; see Figure 1c).

*Shh* is expressed at the posterior margin of chick leg buds for a similar duration to its expression in chick wing buds. Furthermore, it has been demonstrated by treating leg buds with cyclopamine that the positional values that specify the three anterior digits of the chick leg are promoted in response to paracrine Shh signaling in an identical fashion to those that specify the three digits of the chick wing (Towers et al., 2011). However, the positional value for the most posterior digit 4 is promoted in response to autocrine Shh signaling (Figure 2B). Thus, when Shh signaling was attenuated in the chick leg bud by cyclopamine 4 h after onset of *Shh* expression, two toes with digit 1 identities arose—one from the polarizing region, the other from adjacent anterior tissue, while when Shh signaling was attenuated after 8 h, three digits develop, toes with digit 2 identities from the polarizing region and adjacent cells and a toe with a digit 1 identity from cells further away, and so on, until by 16 h, all the antero-posterior positional values in the leg bud have been specified (Towers et al., 2011). These observations show that although it takes slightly longer to specify antero-posterior positional values in the leg compared to the wing, this process is nevertheless accomplished in the early leg bud, and, as in the wing bud, some considerable time elapses before these positional values are interpreted (Figure 2B). It should be noted that, in the *Ozd* chicken mutant, a single digit 1 forms in the leg (Ros et al., 2003).

### Mouse limb

The mouse limb has five digits (1, 2, 3, 4 and 5 in antero-posterior sequence) and digits 2–5 all have three phalanges making them morphologically very similar. Fate maps of the mouse limb polarizing region made by tracing genetically labeled cells that have expressed *Shh* show that the two posterior digits of the mouse limb are entirely derived from the polarizing region, and while there is some contribution to digit 3, the two anterior digits come from cells outside of the polarizing region (Harfe et al., 2004).

*Shh* is expressed at the posterior margin of limb buds of mouse embryos between E9.5–E12.0 (60 h; Zhu et al., 2008, Figure 2C, note expression is between E10-E12.5 in hind-limbs). At E10.5, a graded distribution of Shh across the posterior third of the mouse hind limb bud has been detected by immunohistochemical analysis (Gritli-Linde et al., 2001) in keeping with paracrine Shh signaling specifying antero-posterior positional values as in the chick wing. *Shh* is expressed not only at the posterior margin but also at the anterior of the limbs of several polydactylous mouse mutants (Masuya et al., 1995) consistent with Shh functioning as a polarizing signal in mouse limbs. In contrast, in mouse embryos lacking *Shh* function, the limbs taper toward the tip, and only one digit-like structure (interpreted as digit 1) develops in the hind-limb, while no digits develop in the fore-limb (Chiang et al., 1996). This indicates that Shh is required for the outgrowth of the limb and for the development of structures distal to the elbow/knee in the mouse limb. It should also be noted that in mouse embryos lacking *Shh* function the development of muscles in this distal region of the limb is severely compromised (Kruger et al., 2001) Experiments in which Smoothened activity is deleted specifically in the prospective myogenic cells show that Shh signaling has direct effects on these cells; timing myogenic differentiation, promoting slow muscle differentiation and controlling their migration into the distal part of the limb (Anderson et al., 2012; Hu et al., 2012).

In chick limbs, antero-posterior positional values clearly relate to the identity of a digit that develops in an appropriate position. However, this is not readily observable in the mouse limb due to the difficulties in determining which digits are present in mouse limbs conditionally lacking Shh function. Therefore, there is currently no general consensus about the model which best reflects how positional values are specified in the mouse limb bud. The various models are now discussed below (also see Figures 2C–F).

The first formal model to be proposed for the mouse limb was the temporal expansion model (Harfe et al., 2004). In this model, anterior positional values for digit 2 (and in part for digit 3) are specified in a concentration-dependent fashion by paracrine Shh signaling and then, posterior positional values (for digits 4 and 5) by the duration of autocrine Shh signaling, which is governed by the proliferative expansion and then displacement of cells from the polarizing region (Figure 2C; specification of digit 1 is considered to be Shh-independent in the hind-limb). Consistent with the model, the restriction of paracrine signaling in a *Dispatched* mutant (see later section on Mechanisms of Shh signaling) resulted in loss of one digit, suggested to be digit 2. This model also gained support from the finding that when *Shh* expression was curtailed in the developing mouse limb, this resulted in only three digits developing. The authors identified these digits as being 1, 2, and 3 consistent with the prediction that digits should be lost in a posterior to anterior sequence (Scherz et al., 2007). A particular feature of this model is that it takes considerable time for all the antero-posterior positional values to be specified (Figure 2C), rather than over a short time in the early limb bud. Moreover, it does not take into account promotion through a transitory series of anterior to posterior positional values, which has been demonstrated to occur in the limb buds of the chick.

A later model was proposed by Zhu et al. (2008) based on the results of a more extensive set of experiments, in which *Shh* function was deleted at a series of different stages in mouse limb development. Again, digits were lost with progressively fewer digits developing when Shh function was deleted at earlier and earlier stages. However in this case, the authors suggested that the sequence of digit loss reflects the order in which digits form, with digits that form last being lost first. Thus, for example, they identified the digits in limbs with three digits as being 1, 2, and 4. If their identification of the digits is correct, a posterior digit has formed adjacent to an anterior digit, an outcome not predicted by any previous model. Based on their findings, they proposed a biphasic model for digit patterning—in which Shh has two functions (Figure 2D). In the first phase, Shh specifies positional values across the antero-posterior axis of the very early limb bud, possibly via a concentration gradient, while in the second phase Shh is required to support proliferation and survival of cells that will form the digits (Zhu et al., 2008). It is not clear whether this latter function is a separate direct function of Shh signaling or reflects an essential role of Shh signaling in maintaining sufficient apical ectodermal ridge signaling. According to this model, the resultant digit patterns when Shh function is deleted are due to loss of Shh compromising survival and proliferation of specified digit progenitor cells rather than failure to specify antero-posterior positional values (Zhu et al., 2008). Furthermore, positional values would have to be specified in the early mouse limb bud over a period of approximately 6 h (based on *Ptch1* expression), which suggests that this process is not integrated with growth as in the chick wing.

The ability to observe promotion in chick limbs gives insights into the time required to specify positional values, but in the mouse limb, in which promotion is not readily observed, it is difficult to distinguish between the effects of Shh signaling on specification of positional values and survival and proliferation of the cells that will form the digit condensations. Indeed the time required for digit specification proposed by Zhu et al. does not appear consistent with a model in which antero-posterior positional values are promoted in response to the concentration and/or duration of Shh signaling. However, if one were to take promotion into account, a unifying model can be proposed (Towers et al., 2011). According to this proposal, positional values would be specified early in the mouse limb as suggested in the biphasic model. However, these would only be anterior positional values, which would then be promoted to posterior values by both paracrine and autocrine Shh signaling operating in parallel. Thus, the pattern of digits specified would depend on how far positional values have been promoted at the time at which *Shh* function is deleted in keeping with more conventional models for digit patterning. The digits that develop in the three-digit mouse limb when Shh signaling is curtailed would therefore be predicted to be 1, 2, and 2—a pattern that is readily observed in cyclopamine-treated chick legs (Towers et al., 2011), and occasionally in wings (Pickering and Towers, 2016). However, there are difficulties in applying a promotion model to the specification of digit 5 of the mouse limb as this would imply that it is the last digit of the pattern to be specified (Figure 2F), when in fact it forms before more-anterior digits (Figure 2D, see also discussion in Towers et al., 2011).

## Interaction between positional information and a turing-type mechanism

Although, it has been shown that Shh is the critical signal in controlling development across the antero-posterior axis of the limb, there is evidence that the periodic condensation of cells that will form the digits depends on an underlying Turing type self-organization mechanism independent of graded Shh signaling. In the basic Turing model, diffusible signals—one operating as an inhibitor, the other as an activator—interact to produce the pattern of digits and interdigits. Positional information and self-organization have been presented as competing models of digit development, when in fact the power of both processes operating together has been long recognized (see (Wolpert, 1989) and for original paper on reaction-diffusion (Turing, 1952).

The first indications that such a self-organization mechanism might be involved in limb development came from experiments in which it was shown that recombinant limb buds formed from disaggregated single cells, re-aggregated and placed back in an ectodermal jacket could still form digits (Zwilling, 1964; Pautou, 1973). Indeed, based on this latter study, one of the first computer simulations of limb development was developed (Wilby and Ede, 1975). Further experiments showed that when recombinant limbs were made from chick mesenchyme cells from the anterior halves of early chick leg buds, which would not include a polarizing region, and which would not normally give rise to digits, two or three morphologically similar digit-like structures developed (Hardy et al., 1995; Elisa Piedra et al., 2000). When a polarizing region was grafted into such recombinant limbs, however, the digits that developed had recognizable identities (MacCabe et al., 1973). These experiments elegantly revealed that positional information and self-organization are integrated in limb development. There is evidence that a self-organization mechanism also operates in mouse limb buds, as the limbs of mutant mouse embryos in which the Shh signaling pathway is non-functional have many morphologically similar digits (Litingtung et al., 2002; te Welscher et al., 2002; see Section–Measurement of Shh concentration and duration of signaling). Indeed, recent studies in the mouse limb have suggested that this mechanism is based on WNT signals acting as inhibitors and BMP signals as activators, that together, converge on the transcription factor Sox9 to generate a repeated series of digit condensations (Raspopovic et al., 2014).

Since digits 2–5 have similar morphologies in the mouse limb, particularly in regard to phalangeal count, one proposal is that self-organization plays a dominant process (Delgado and Torres, 2016). This scenario could for account for difficulties in applying a positional information model to the five digits of the mouse limb. Moreover, a recent study on developing chick wings has revealed how positional information and self-organization can interact and this could be relevant to understanding how the mouse digit pattern is specified. If chick wing buds are treated with cyclopamine under conditions in which the promotion of antero-posterior values is truncated, a series of morphologically similar digit 2s in a pattern 1-2-2-2 can develop by self-organization (Pickering and Towers, 2016). It should be noted that the digit 2s were not of identical morphologies and sizes suggesting other factors control these finer aspects of development. In wings with multiple digit 2s, the most-posterior of these digits arises from cells of the polarizing region. An interpretation of these findings is that antero-posterior expansion mediated by a posteriorly extended apical ectodermal ridge has enabled a small pool of cells specified with the same positional value to produce a series of digit 2s by self-organization (Pickering and Towers, 2016). In extrapolating these data to the mouse limb, it has been suggested that a similar mechanism could account for the patterning of digits 1 through to 4 (Pickering and Towers, 2016; Figure 2F). In addition, the apical ectodermal ridge of the mouse limb completely overlies the polarizing region (Pickering and Towers, 2016), and an intriguing suggestion is that this could enable the cells of the polarizing region to expand sufficiently to give rise to two digits (4 and 5) by self-organization (Figure 2F; Pickering and Towers, 2016). The specification of the same positional value during mouse limb development could occur if cells become refractory to the levels/duration of Shh signaling at a certain point (Figure 2F). In support of such a mechanism operating in the mouse, there is not a simple linear relationship between position and level of positive Shh signaling in the limb bud as expected in a classical positional information model (Ahn and Joyner, 2004). However, even though mouse digits 2–5 are morphologically similar, it is clear that they still have different identities, with the cells of digit 4 being characterized by having many more receptors for both testosterone and estrogen than digit 2 thus determining the sexual dimorphism in digit length (Zheng and Cohn, 2011). Indeed, digit 5 in particular, has quite a different morphology to the other digits. Taken together, even if the cells that give rise to mouse digits 2–5 are specified with the same positional value that is interpreted so that they have the same phalanx number, other factors operate to give the digits their individual morphologies and hence identities. Additional support for a model in which loss of Shh signaling can increase digit number and also result in posterior digits developing with anterior traits has been provided by work on the fore-limbs and hind-limbs of the amphibian *Xenopus tropicalis*. Inhibition of Shh signaling at a series of developmental stages resulted in fore-limbs occasionally developing with five digits rather than four (Stopper et al., 2016). In addition, hind-limbs often developed terminal claws on all five digits whereas in normal development claws are only present on digits 1, 2, and 3. Additional work is required to determine if other characteristics of these posterior digits are anteriorised such as phalange number.

The work of Pickering and Towers further highlights the complex relationship between the polarizing region and the apical ridge already mentioned (Niswander et al., 1994), and the importance of short-range reciprocal signaling between these structures in the formation of posterior digits in particular as observed in the mouse limb (Zuniga et al., 1999; Bouldin et al., 2010). Thus, in the chick wing, Shh signaling inhibits the overlying apical ridge and the polarizing region fails to produce digits, yet in the mouse limb, the overlying apical ridge is less sensitive to Shh signaling than in the chick wing (see also (Bouldin et al., 2010), and in persisting posteriorly, allows two digits to form—the chick leg appears to have an intermediate relationship allowing one digit to form. Such dynamic interplay between the polarizing region and apical ridge could have contributed to patterns of posterior digit loss during limb evolution (see Section on Evolutionary aspects of Shh signaling in the limb).

## Mechanisms of Shh signaling

As indicated in the models outlined above, positional values in developing limbs are specified by paracrine Shh signaling, in which Shh acts as a long-range graded signal and in a concentration/time dependent fashion, or by the duration of autocrine Shh signaling. Therefore, the crucial questions are how a graded distribution of Shh arises, how the range of Shh signaling is controlled and how cells measure the concentration of Shh and the duration of Shh signaling.

### Long-range Shh signaling and gradient formation

Studies in developing mouse limbs have revealed general mechanisms that modulate the distribution of Shh protein in tissues. One factor is the addition of lipids. Following its autocatalytic conversion, Shh is secreted by cells as a modified form of ShhN with cholesterol added at the C-terminus and a palmitoyl group (as part of a thiol ester) at the N-terminus (known as ShhNp; p indicating that ShhN is processed; reviewed (Lee et al., 2016). In limb buds of mouse embryos in which the C-terminal processing domain of *Shh* is conditionally deleted so that the polarizing region produces ShhN instead of ShhNp, ShhN spreads further across the limb bud and additional digits develop anteriorly (Li et al., 2006). It should be noted that previous analyses also suggested that cholesterol modification extends the range of paracrine Shh signaling. Thus, mice limbs expressing ShhN that lacks cholesterol failed to form digits 2 and 3 (Lewis et al., 2001) consistent with a role for paracrine Shh signaling in specifying these digits (Harfe et al., 2004). Other data however are consistent with cholesterol modification restricting the spread of Shh. Thus, mice deficient in SREBP-2 that encodes a sterol regulatory element binding protein that regulates cholesterol production failed to up-regulate *Ptch1*, consistent with impaired Shh transport (Vergnes et al., 2016). Similar studies on mutant mice that are unable to palmitoylate Shh show that this modification is essential for long range signaling (Chen et al., 2004). Intriguingly, cholesterol has also recently been shown to be the endogenous activator of Smoothened (Huang P. X. et al., 2016). Because cholesterol plays such important roles in Shh signaling, changes in the availability of cholesterol can impact on the development of the limb and might explain the subtle alterations in the spacing of the digits that have been observed in the limbs of mice with a mutation in a gene encoding a protein required for cholesterol metabolism (Schmidt et al., 2009) and in the limbs of rat embryos treated with triparanol, an inhibitor of cholesterol biosynthesis (Gofflot et al., 2003). The membrane protein Dispatched1 is required for paracrine signaling by cholesterol–modified Shh (Tian et al., 2005). The restriction of the spread of the ligand in a *Dispatched1* mouse mutant resulted in the loss of a digit, which was interpreted as being digit 2, and as already mentioned, provided crucial evidence for the temporal expansion model (Harfe et al., 2004).

Another mechanism that influences the range of Shh signaling is the binding of Shh to cell surface and extracellular proteins. A generic response to Shh in all tissues is transcriptional up-regulation of genes encoding cell surface proteins such as *Ptch1* and *Hhip* that bind Shh. The resultant increase in their expression in response to Shh creates negative feedback loops, that not only limit the spread of Shh by sequestering it at the cell surface, but also, in the case of Ptch1, because it inhibits Smoothened activity, dampens activation of the Shh pathway. In mice in which *Ptch1* is conditionally inactivated in the limbs (Butterfield et al., 2009), and therefore the signaling pathway is activated independently of Shh, the hind-limbs have extra digits, but the fore-limbs have fewer digits. This difference between hind-limbs and fore-limbs appears to be due to the timing of activation of the signaling pathway, which is earlier in the mutant fore- limbs (Zhulyn et al., 2014).

In contrast to *Ptch1* and *Hhip1*, the genes *Cdo* (*CAM-related/downregulated by oncogene*), *Boc* (*brother of Cdo*) and *Gas1* (*growth arrest specific 1*) encoding membrane associated proteins that bind Shh, are expressed in the anterior region of early limb buds and their expression is negatively regulated by Shh. Analysis of limb development in single or double mouse mutants suggest that Gas1 and Boc sustain paracrine Shh signaling at a distance from the polarizing region (Allen et al., 2007). ShhNp can also bind to heparan sulfate proteoglycans and the distribution of these and other extracellular proteins in the developing limb will affect the distribution of Shh. In *Drosophila*, the hydrolase notum that cleaves glypicans, a sub-family of heparan sulfate proteoglycans, promotes high-level Hh signaling in the wing. Interestingly, in the chick wing bud, *Notum* was identified in microarray experiments as being downstream of Shh signaling (Bangs et al., 2010), suggesting possible functional conservation.

One way in which Shh could spread across the limb bud is by diffusion (see Muller et al., 2013, for discussion on mechanisms of morphogen transport), although it has been questioned whether simple diffusion would be a sufficiently robust mechanism to generate a stable concentration gradient (Kerszberg and Wolpert, 2007). Mathematical modeling however showed that specification of positional values for the three digits of the chick wing can be simulated by simple diffusion of Shh from the polarizing region (Woolley et al., 2014). In the model, based on the results of (Drossopoulou et al., 2000), Shh specifies the initial size of the domain that will give rise to the digits and then provides positional information. The model incorporates promotion of positional values in a dose-dependent fashion over the observed time frame in a growing domain of the correct dimensions as determined experimentally (Towers et al., 2008). The model can be extended successfully to the specification of the positional values in the chick leg, even though digit 4 arises from the polarizing region. However, it is unclear whether Shh levels in the polarizing region could reach the predicted concentration required to specify digit 4 (assumed to be double that required to specify digit 3) and whether indeed there is a simple graded response to Shh signaling in the leg. It is therefore more plausible that digit 4 is specified by length of time that cells express *Shh*. The model cannot however be extended further to simulate easily specification of the fifth digit of the mouse limb.

Live imaging of chick wing buds showed that Shh can be transported along the external surface of specialized filopodia (similar structures in insects are called cytonemes). These filopodia extend up to 150 microns away from the polarizing region and a similar distance away from the receiving cell (Sanders et al., 2013) equating to about 300 microns, the initial size of the chick wing digit-forming field (Vargesson et al., 1997; Towers et al., 2008). Thus, direct cell-cell contacts can span the required range of Shh signaling. Furthermore, Boc and Cdo have been visualized in discrete microdomains on a subset of filopodia extending from Shh-responding cells. However, it is not clear whether this transport mechanism could produce robust graded signaling and indeed whether filopodia are required. The involvement of filopodia could however explain the apparently anomalous finding that grafts of cells expressing a membrane-tethered form of Shh (generated by fusing the integral membrane protein CD4 to the C-terminus of ShhN) can duplicate digits in the chick wing (Yang et al., 1997).

### Measurement of Shh concentration and duration of signaling

It has been proposed that limb bud cells respond to paracrine Shh signaling in a concentration dependent fashion although length of exposure to the Shh signal also plays a role. So how do cells measure the concentration of Shh? The mechanism depends on the Shh-dependent processing of full-length Gli proteins, which act as transcriptional activators; in the absence of Shh signaling, Gli proteins are processed to short forms, which act as transcriptional repressors (reviewed in Lee et al., 2016). In normal chick and mouse limb buds, anterior cells not exposed to Shh contain high levels of Gli repressor, while in the posterior region of the limb, there is a gradient in the ratio of Gli activator/Gli repressor, higher posteriorly than anteriorly, reflecting the response to the Shh gradient across this part of the limb (Wang et al., 2000). There are three *Gli* genes, *Gli1, Gli2*, and *Gli3* with the protein encoded by *Gli1* acting exclusively as an transcriptional activator as it does not undergo processing into a repressor form. While functional inactivation of *Gli1* and *Gli2* in mice has little effect on limb development (Mo et al., 1997; Park et al., 2000), when *Gli3*, the major contributor to transcriptional repression, is functionally inactivated, *Shh* is expressed anteriorly and several additional morphologically similar digits form anteriorly while posterior digits are less affected (Wang et al., 2000). Unexpectedly, the limbs of *Gli3* and *Shh* double knockout embryos are identical to the *Gli3*^−/−^ limb buds showing that the function of Shh in the limb is to relieve repression by Gli3 and allow a patterned set of digits to develop from the posterior part of the limb (Litingtung et al., 2002; te Welscher et al., 2002). In the mouse limb, the gradient of Gli3 activity could only specify at most digits 1, 2, and 3 because *Gli3* is not expressed in the polarizing region itself (Buscher and Ruther, 1998). Instead the initial response to autocrine Shh signaling would have to be mediated by Gli2, and consistent with this hypothesis, removing the function of *Gli2* in a *Gli3* mutant background, thus effectively inactivating all Gli function, results in the digits appearing morphologically similar (Bowers et al., 2012). This suggests that Gli3 mediates the response of cells in the limb bud to paracrine Shh signaling and Gli2 to autocrine Shh signaling. It should also be noted that the digits that form in single *Gli3*^−/−^ mouse limbs (and also in compound *Shh*^−/−^*/Gli3*^−/−^ mouse limbs) are thinner and more closely spaced together than in normal limbs, suggesting that Gli3 plays a role in regulating the digit period (Sheth et al., 2012, see section Interaction Between Positional Information and a Turing-type Mechanism). 5′Hoxa/d function also seems to be involved since the progressive titration of *5*′*Hox* genes in the *Gli3*^−/−^ background increases digit number and decreases the digit period still further (Sheth et al., 2012).

Surprisingly, chemical mutagenic screens to identify mutations causing polydactyly in mouse identified genes required for formation and functioning of primary cilia (Huangfu et al., 2003; Weatherbee et al., 2009; Ashe et al., 2012). In such mutants, many morphologically similar digits develop and this is because Gli processing takes place on primary cilia in vertebrate cells. Thus, absence of cilia is equivalent to functional inactivation of all three Gli genes. The classical chicken mutant, *talpid3*, with a range of defects including polydactylous limbs (Ede and Kelly, 1964) was found to have a mutation in a gene encoding a centrosomal protein required for formation of a primary cilium (Davey et al., 2006), and functionally inactivating the *talpid3* gene in a mouse limb, leads to the development of many morphologically similar digits (Bangs et al., 2011). Another chicken mutant, *talpid2*, with the same range of defects including polydactylous limbs, was found to have a mutation in a gene encoding another ciliary protein—C2CD3 (Chang et al., 2014).

For autocrine Shh signaling, the duration of signaling is the most important parameter. Timing appears to be a general way of specifying positional values, but how cells in embryos measure time is little understood. Interestingly, a timing mechanism involving a cell cycle clock has been proposed to specify proximo-distal positional values in the chick wing bud (Saiz-Lopez et al., 2015), although the most proximal positional values may be specified by retinoic acid signaling (Cooper et al., 2011; Rosello-Diez et al., 2011). The molecular nature of intrinsic timers is currently unknown and presents a widespread problem in developmental biology.

## Initiation of *Shh* expression

A key discovery in understanding how *Shh* expression is localized to the posterior margin of the limb bud was identification of a *cis*-regulatory element that controls limb-specific expression (Lettice et al., 2002). Analysis of *Sasquatch*, an insertional mouse mutant with limb polydactyly, in which *Shh* was expressed anteriorly as well as posteriorly in the limb, showed that the exogenous DNA construct had serendipitously disrupted an enhancer (Sharpe et al., 1999). This 1.7 Kb enhancer, which has become known as the ZRS (zone of polarizing activity regulatory sequence), is unexpectedly located in intron 5 of the *LMBR1* (*limb region 1*) gene, which is almost 1 MB upstream of the promoter of the *Shh* gene. It is still not clear why insertion of the transgene into this particular region of the ZRS in *Sasquatch* leads to anterior *Shh* expression in the limb bud. In contrast, deletion of the entire ZRS region in mouse embryos results in loss of *Shh* expression in the limb buds resulting in limb truncations similar to those found in mouse embryos lacking *Shh* function (Sagai et al., 2005). It should be noted however, that the many other defects seen in mouse embryos lacking *Shh* function, which reflect the widespread functions of Shh signaling in organogenesis, are not present in the mouse embryos in which the ZRS is deleted.

The ZRS is of general interest as an example of a long-range enhancer—a cluster of three similar long-range enhancers also regulates *Shh* expression in the epithelial linings of the pharynx, the lung and the gut respectively (Sagai et al., 2009). 3D FISH and chromatin configuration assays showed close associations between the ZRS and the *Shh* locus in mouse limb bud cells compared to cells from other tissues (Amano et al., 2009). Curiously, transcriptional activity was not seen in all polarizing region cells suggesting that the cells may express *Shh* in pulses. One possibility is that *Shh* is expressed periodically during the cell cycle. In support of this, *Shh* expression is lost in chick wing buds treated with aphidicolin—an inhibitor of progression through S-phase (Ohsugi et al., 1997). More recently FISH and chromatin configuration assays together with super-resolution microscopy have revealed that the *Shh* locus loops out of its chromosome territory to make contacts with the ZRS in polarizing region cells in the mouse limb bud at the time *Shh* expression is activated (Williamson et al., 2016).

The ZRS provides an excellent reference point for deciphering the gene network that controls *Shh* expression in the limb and contains binding sites for the transcription factors, Hand2 (heart and neural crest derivatives 2; (Galli et al., 2010) and 5′ Hoxd proteins. The genes encoding these transcription factors are expressed in the posterior region of the early limb bud and when they are deleted in the mouse limb, *Shh* is not expressed. Conversely, when *Hoxd13* is expressed throughout the mouse limb bud, there is an ectopic *Shh* domain and polydactylous limbs result (Zakany et al., 2004).

Expression of *Hand2* and *Hoxd* genes is restricted to the posterior part of mouse limb buds by Gli3. In the mouse fore-limb-forming region, *Hand2* expression is also repressed anteriorly by the *Hox5* paralogous group genes (Xua et al., 2013), while *Hand2* expression in the posterior region of the fore-limb-forming region is dependent on the *Hox9* paralogous group genes, thus providing antero-posterior polarity prior to the transcriptional activation of the *Shh* gene (Xu and Wellik, 2011). Recently, it has emerged that GATA family transcription factors also contribute to supressing anterior expression of *Shh* (Kozhemyakina et al., 2014) as conditional removal of *Gata4/6* in limbs of mouse embryos results in pre-axial polydactyly. Two distinct mechanisms have been proposed. One is that GATA transcription factors in complex with FOG co-factors bind directly to the ZRS enhancer while the other is that GATA6 may interact directly with GLI3 to promote repression of the vertebrate Hedgehog pathway and this may explain the formation of an additional anterior digit in the hindlimb (Hayashi et al., 2016).

*Shh* expression in the polarizing region is also controlled by FGF signaling from the apical ridge and FGF signaling has been shown to regulate the expression of the genes encoding the ETS translocation variant transcription factors ETV4 and ETV5. The genes encoding these transcription factors are expressed beneath the entire extent of the apical ectodermal ridge and suppress *Shh* expression outside of the polarizing region. These ETV transcription factors bind directly to sites in the ZRS. In the polarizing region, posteriorly expressed ETS1/GABPα binds to other sites in the ZRS and over-rides this inhibition and allows expression of *Shh* (Lettice et al., 2012). Wnt7a signaling from the dorsal ectoderm also contributes to controlling *Shh* expression but the mechanism is not yet known (Yang and Niswander, 1995).

The activity of the ZRS not only determines the location of cells expressing *Shh* in the developing limb bud but also the size of the *Shh* expression domain. In addition, an autoregulatory mechanism has been discovered in which Shh controls the number of polarizing region cells by regulating the size of the posterior necrotic zone (Sanz-Ezquerro and Tickle, 2000) via BMP2 signaling (Bastida et al., 2009) Taken together these mechanisms have the crucial function of controlling the levels of Shh signaling.

Lastly, retinoic acid derived from the flank also appears to be required for initiating *Shh* expression in limb buds. *Shh* expression is greatly reduced in the limb buds of vitamin A deficient quails (Stratford et al., 1999) and in chick wing buds following treatment with inhibitors of retinoic acid synthesis (Stratford et al., 1996). Mouse embryos in which a gene encoding an enzyme that generates retinoic acid was functionally inactivated died early and lacked fore-limbs. When these embryos were provided with retinoic acid so that development can proceed further, *Shh* was not restricted posteriorly in the rescued fore-limb buds suggesting that retinoic acid plays a role in determining antero-posterior polarity prior to activation of *Shh* expression (Niederreither et al., 2002; Zhao et al., 2009).

## Termination of *Shh* expression

The failure of the positive feedback loop between the polarizing region and the apical ectodermal ridge has been proposed to terminate the duration of *Shh* expression in the chick wing. In this model, Shh up-regulates *Grem1* by paracrine signaling, but cells displaced from the polarizing region by proliferative expansion are then unable to up-regulate *Grem1* (the apical ridge maintenance factor; Scherz et al., 2004). This is proposed to create a tissue barrier that results in Shh being no longer able to up-regulate *Grem1* at a distance, leading to de-repressed BMP signaling suppressing *Fgf4* expression in the apical ectodermal ridge, that in turn, leads to loss of *Shh* expression in the polarizing region (Scherz et al., 2004). Tbx2 is proposed to be the factor that suppresses the posterior up-regulation of *Grem1* in and around the polarizing region (Farin et al., 2013). In the absence of Tbx2, *Grem1* expression expands posteriorly resulting in prolonged *Shh* expression and extra tissue growth indicated by the bifurcation of digit 4. It is unclear why this only occurs in the hind-limbs of these *Tbx2* knockout mice. An alternative model for the mouse limb is that increased FGF signaling inhibits *Grem1* expression leading to termination of the feedback loop (Verheyden and Sun, 2008).

A clock linked with the cell cycle has also been shown to be involved in timing the duration of *Shh* expression in the polarizing region of the chick wing bud with the clock being set once retinoic acid concentrations fall below a certain level. Thus, tissue transplantation experiments have shown that the chick wing polarizing region intrinsically times the duration of *Shh* expression irrespective of the extrinsic signaling environment (Chinnaiya et al., 2014). Indeed, *Shh* expression has been shown to terminate on time if the separation of *Grem1* and *Shh* expressing cells is prevented (Towers et al., 2008). Furthermore, the inhibition of Shh signaling with cyclopamine in the chick wing leads to the premature loss of *Shh* expression in the presence of an *Fgf4*-expressing apical ectodermal ridge and *Grem1* expression extending into the posterior part of the wing bud, thus suggesting that Shh autoregulates its own transcription in the polarizing region (Pickering and Towers, 2016). The mechanism by which this is achieved has not yet been elucidated.

## Response to Shh signaling in the limb

Many studies have provided information about the expression of individual genes that are affected by Shh signaling in the limb. For example, changes in gene expression have been observed in chick limb buds treated with Shh or cyclopamine, and in mouse limb buds in which *Shh* or *Gli3* is functionally inactivated, or in which Gli3 processing does not occur, e.g., mutants with defective cilia. Microarray analyses have been carried out in both chick and mouse limbs (Vokes et al., 2008; Bangs et al., 2010). It has been estimated from one microarray study that 10% of the genes expressed in the early limb bud (about 1,000 genes) are downstream of Shh signaling (Bangs et al., 2010). Putative direct targets of Gli3 repression have been identified by ChIP seq analysis of limb bud nuclear extracts using transgenic mice expressing a tagged form of the Gli3 protein (Vokes et al., 2008). Further analysis has involved RNAseq (Lewandowski et al., 2015).

Analysis of this information has begun to uncover the gene regulatory network underlying the response to the Shh signaling pathway in the limb in addition to the generic suite of genes that encode proteins that enable or modulate Shh signaling. The genes in the network include those that are expressed posteriorly either due to positive regulation by Shh or because Shh relieves Gli3 repression; also those that are expressed anteriorly either due to negative regulation by Shh or because they are downstream of Gli3 repression (Bangs et al., 2010). A study involving analysis of gene expression patterns in the limb buds of *Shh*^−/−^*, Gli3*^−/−^ double mouse mutants indicated that the expression of nearly all the putative Gli target genes identified by ChIP seq in the posterior mesenchyme of E10.5 mouse limb buds depends on Gli repressor activity rather than Gli activator activity (Lewandowski et al., 2015).

One generic class of potential target genes already mentioned comprises genes encoding cell cycle regulators such as *N-myc* and *Cyclin D1* that are predominantly expressed posteriorly and *Cyclin D2* that is expressed in the polarizing region, and that are likely mediate the effects of Shh on proliferation (Towers et al., 2008; Welten et al., 2011). Shh has also been shown to promote vascularisation of the chick wing bud via regulating expression genes encoding pro-angiogenic factors such as VEGF (Davey et al., 2007). There is evidence in the mouse limb, that transcription factor genes including 5′ genes in the *Hoxa* and *Hoxd* clusters, *Sall1*, and *Tbx2/Tbx3* are putative direct targets of Shh and would be predicted to encode the positional information conferred by the autocrine/paracrine Shh signaling (Vokes et al., 2008). Experiments with cultured mouse limb buds suggest that Shh signaling is required for robust and continued expression of 5′members of the *Hoxd* cluster (Panman et al., 2006; Lewandowski et al., 2015) while mis-expression of *Tbx2* and *Tbx3* genes in the chick leg bud in the embryo has been reported to change digit identity (Suzuki et al., 2004).

Other putative direct Gli3 targets are genes involved in BMP signaling; *Gremlin* encoding the apical ridge maintenance factor and *Bmp 2* expressed together with *Bmp7*, in the posterior region of the early limb bud (Vokes et al., 2008). There is a close relationship between *Shh* and *Bmp2* expression elsewhere in vertebrate embryos, which is also conserved in *Drosophila*. For instance in the *Drosophila* wing imaginal disc, Hh secreted from the posterior compartment induces expression of the *Bmp2* orthologue, *Dpp*, that encodes a long range signaling molecule regulating position-dependent expression of transcription factors such as *Spalt* and *Omb*, orthologues of *Sall1* and *Tbx2/3* respectively. Experiments in chick wing buds show that Bmp-soaked beads placed at the anterior margin of a chick limb do not induce digit duplications (Drossopoulou et al., 2000). However, when a bead soaked in a BMP antagonist was implanted at the anterior margin of the wing bud following implantation of an Shh-soaked bead, a series of morphologically similar digits developed anteriorly suggesting that BMP signaling is involved in digit promotion (Drossopoulou et al., 2000). In chick leg buds, BMP signaling is graded across the tip of the bud at the stage at which the digit condensations form in the so-called phalanx-forming region (PFR—Suzuki et al., 2008). Grafting interdigital tissue to different positions between digit condensations and manipulating BMP signaling alters the morphology of the digits in terms of phalange number suggesting that it is BMPs produced by interdigital regions that are directly responsible for realizing digit-specific morphology (Dahn and Fallon, 2000). Recently, evidence has been presented that interdigital signaling may also be involved in regulating the morphogenesis of the digit condensations in mouse limbs (Huang B. L. et al., 2016).

## Clinical aspects of Shh signaling in the limb

The increasing understanding of the molecular basis of antero-posterior pattern formation has led to insights into congenital malformations that affect the limb. Unsurprisingly, defects in Shh function have been found to underlie several inherited disorders. In particular, these include polydactyly: pre-axial polydactyly in which additional digits arise from the thumb-side of the hand, and post-axial polydactyly in which the additional digits arise from the little finger-side (Biesecker, 2011). Often these conditions are associated with syndactyly (fusion of the soft tissues between the digits).

Alterations in the coding sequence of the *SHH* locus are not known to form the basis of any congenital malformation of the limb—presumably because such lesions are not compatible with the development of other tissues. However, point mutations in the ZRS enhancer that would be predicted to lead to ectopic *SHH* expression specifically in the limb bud are found in human patients with pre-axial polydactyly type 1 (PPD1—OMIM 174400) and triphalangeal thumb polysyndactyly syndrome (TPTPS OMIM 174500) (see review Hill and Lettice, 2013). In TPTPS, additional digits can arise post-axially as well as pre-axially, suggesting that the normal regulation of *SHH* expression at the posterior margin of the limb is also perturbed. It remains to be determined how these point mutations affect the regulation of endogenous *SHH* expression. One possibility is that the levels and/or duration of *SHH* expression are increased and these lead not only to an additional digit pre-axially but also to overgrowth of the polarizing region and its subsequent development into additional post-axial digits—perhaps by self-organization (see section on Interaction between positional information and a Turing-type mechanism). A point mutation at a particular position in the ZRS is associated with Werner mesomelic syndrome in which there are distal arm and leg bone defects in addition to extra digits (VanderMeer et al., 2014). Unexpectedly, duplications of the ZRS have also been reported in individuals with TPTPS as well as the related condition Haas-type polysyndactyly (OMIM 186200). Microduplications of the ZRS have also been detected in patients with Laurin-Sandrow syndrome OMIM 13750); the limb phenotype of these patients overlaps with the Haas-type polysyndactyly phenotype but can be distinguished by mirror-image polysyndactyly of the feet and duplication of the fibula (Lohan et al., 2014). In contrast, patients with a deletion involving exon 4 and portions of introns 3 and 4 of the *LMBR1* gene, a region distinct from the ZRS, have a condition known as acheiropodia (OMIM 200500) in which elements distal to the elbow/knee fail to form in all four limbs. This condition not only resembles the phenotype of the limb buds of mouse embryos lacking Shh function but also that of the limbs of *Ozd* mutant chickens in which it has now been shown that a large part of the ZRS sequence is deleted (Maas and Fallon, 2004). Inborn errors in cholesterol metabolism can lead to limb anomalies, as might be expected given the importance of cholesterol in Shh signaling as already discussed. For example, post-axial polydactyly is found in patients with Smith-Lemli-Opitz syndrome (OMIM 270400) in which a mutation deactivates the function of 7-dehydrocholesterol reductase, which is the final enzyme in the metabolic pathway that generates cholesterol. Post-axial polydactyly is also seen at low frequencies in patients with other syndromes in which cholesterol biosynthesis is altered (Gofflot et al., 2003). Why post-axial polydactlyly occurs however is not clear.

Defects in the response to Shh signaling are found in syndromes that include polydactyly. For instance, the Pallister-Hall (OMIM 146510, Hill et al., 2007) and Grieg Cephalopolysyndactyly (OMIM 175700- Kalff-Suske et al., 1999) syndromes present with pre-axial and post-axial polydactyly and are caused by mutations in the *GLI3* gene. The effects of these mutations are likely due to the de-repression of the Shh signaling pathway in the anterior part of the limb. Since the processing of full-length Gli3, occurs in primary cilia, syndromes known as ciliopathies, in which cilia function/structure is compromised, include polydactyly as part of their spectrum of defects—examples being, Bardet-Biedl syndrome (BBS–OMIM 209900, Forsythe and Beales, 2013) and Meckel-Gruber syndrome (OMIM 249000, Shaheen et al., 2013). Recently mutations in the *TALPID3* gene, required for formation of cilia have been discovered in patients with Joubert syndrome (OMIM 21330) although these patients rarely show limb defects (Roosing et al., 2015; Stephen et al., 2015). Homozygous mutations in the *TALPID3* gene have however been found in families affected by lethal ciliopathies associated with polydactyly (Alby et al., 2015), phenotypes more akin to those of the homozygous chicken mutants already mentioned in which the *talpid3* gene was first identified.

Several clinical conditions are associated with mutations in putative gene targets of Shh signaling in the limb (see previous section on Response to Shh signaling, also reviewed Pickering and Towers, 2014). *Sall1* encoding a transcription factor is expressed in the posterior region of the early chick and mouse limb buds but more widely at the base of the digital plate at later stages (Buck et al., 2001; Fisher et al., 2011). Mutations in *SALL1* that produce a truncated protein with dominant negative activity have been detected in patients with Townes-Brockes syndrome characterized in the limb by pre-axial polydactyly and triphalangeal thumb (Kohlhase et al., 1998). A transgenic mouse model in which a truncated SALL1 protein is produced mimics the human limb phenotype (Kiefer et al., 2003). Inactivating mutations in the gene encoding the transcription factor Tbx3, which is expressed at high levels in stripes at both anterior and posterior margins of early chick and mouse limb buds (Tumpel et al., 2002; Emechebe et al., 2016) are seen in patients with Ulnar-mammary syndrome (OMIM 181450); the defects affect the development of posterior structures in the upper limb and include missing ulna, missing posterior digits and post-axial polydactyly. The same limb phenotype is seen in mouse *Tbx3* mutant embryos (Davenport et al., 2003; Emechebe et al., 2016). Finally mutations in *HOXD13* are associated with many clinical conditions in which there are digital abnormalities including polydactyly, syndactyly (fused digits) and brachydactly (short digits). *Hoxd13* is another putative gene target of Shh signaling identified in the mouse limb and is expressed in the posterior region of early chick and mouse limb buds and then throughout the digital plate at later stages (Nelson et al., 1996). A complex spectrum of mutations in *HOXD13*-polyanaline tract expansions, truncating mutations and point mutations leading to amino acid substitutions have been identified (reviewed Goodman, 2002). *Hoxd13* is likely to have several roles in digit development and the challenge is to understand how a particular genetic change leads to a particular phenotype.

## Shh signaling and limb regeneration

Adult urodele amphibians (newts and salamanders) can regenerate their limbs after amputation. *Shh* signaling occurs in adult urodele limbs during regeneration and understanding how *Shh* expression is activated in these adult tissues may be relevant in the context of stimulating growth and repair of tissues in damaged limbs. Following amputation of a newt limb, a mound of undifferentiated cells called the blastema forms at the stump surface and proliferation of blastemal cells replenishes the missing limb structures. *Shh* is expressed in posterior part of the newt limb blastema recapitulating embryonic expression in the limb bud (Imokawa and Yoshizato, 1997), and when regenerating salamander limbs were treated with cyclopamine, only one digit-like structure formed—similar to hind-limbs of *Shh* mutant mice (Chiang et al., 1996). Recently, it has been demonstrated that *Shh*, which is expressed in the posterior part of the salamander blastema is part of a reciprocal feedback loop via *Grem1* and *Fgf8* that are expressed in the anterior part of the blastema (Nacu et al., 2016). This feedback loop is required for outgrowth of the blastema and closely recapitulates the epithelial-mesenchyme signaling network that drives embryonic limb development. The demonstration that two signals, which can act at a distance—Shh and Fgf8—drive limb regeneration is at odds with a long standing model in which direct cell-cell interactions stimulate intercalary growth to even out disparate positional identities between anterior and posterior parts of the blastema (French et al., 1976). The size of the limb blastema is about 10 times that of embryonic limb buds, therefore it is not clear whether these signals could indeed act over the large distances involved.

Fate maps of the blastema showing which cells give rise to the digits and experiments addressing timing of specification of antero-posterior positional values could give important insights into whether digit regeneration is comparable to embryonic development. One possibility is that cells within a blastema maintain memory of their position along the antero-posterior axis and restore missing structures by a timing mechanism linked to proliferation. Evidence for such a cellular memory based on epigenetic modifications has been obtained in regenerating limb buds of Xenopus embryos (Hayashi et al., 2015). A timing model would dispense with difficulties in scaling long range gradients over considerable distances to restore missing positional values during regeneration and the role of Shh and Fgfs would be to maintain the outgrowth and the width of the blastema. It would also be useful to know the fate of polarizing region cells from embryonic urodele limb buds in adult limbs and regenerating limbs.

Unlike urodeles, anuran amphibians can only regenerate their limbs during embryonic stages. Interestingly, increased methylation of the ZRS enhancer during *Xenopus* development correlates with reduced capacity to regenerate the limb in the adult suggesting that epigenetic mechanisms limit this process by preventing re-expression of *Shh* (Yakushiji et al., 2007).

## Evolutionary aspects of Shh signaling in the limb

The ZRS element located in the fifth intron of *Lmbr1* gene that drives limb-specific *Shh* expression is well conserved at the sequence level in many vertebrates. The ZRS is an excellent candidate for evolutionary modifications that have resulted in changes in limb morphology because the rich diversity of limb morphologies could have evolved without affecting other features of the body plan. In support of this, mutations in the ZRS at a conserved ETS1 binding site in pythons have been described that appear to be responsible for the early loss of *Shh* expression and subsequent failure of limb bud outgrowth (Kvon, 2016; Leal, 2016). CRISPR/CAS9 gene editing approaches, in which the mouse ZRS was replaced by the python ZRS sequence, resulted in limb truncations similar to those obtained upon the complete removal of *Shh* function in the mouse limb (Kvon, 2016). As in pythons, *Shh* fails to be up-regulated in the hind-limbs of the spotted dolphin and is associated with reduced outgrowth, although the molecular basis of this has not been examined (Thewissen et al., 2006). Many described ZRS mutations to date, however, result in ectopic expression of *Shh* in the anterior part of the limb, and therefore the development of additional digits as in domesticated animals; for instance, Dorking's (Bouldin and Harfe, 2009) and Silkie chickens (Dunn et al., 2011) have an additional anterior digit in the leg and dogs and cats (notably Hemingway cats) have extra anterior digits in their fore-paws (Lettice et al., 2008).

Limbs with more than five digits have not been selected for during evolution suggesting there is little benefit in increasing digit number. Interestingly, the limbs of the earliest Devonian tetrapods such as *Acanthostega* and *Icthyostega* had up to eight digits (Clack, 2002). The mechanism by which such digit patterns would have been specified is of considerable interest. In having several digits, the limbs of such tetrapods superficially resemble the limbs of mouse *Gli* mutants, which have many digits that form by self-organization. However, the digits in these Devonian tetrapods display differences in phalangeal number suggestive of antero-posterior positional values specified by Shh in the early limb bud. Once pentadactyly was established in tetrapods, this has remained the basic plan, although occasionally limbs with so-called “sixth digits” have evolved. These sixth digits are in fact, adaptations of other limb bones, such as the overgrown wrist bone in the case of the mole's “paddle-like” limb (Mitgutsch et al., 2012). The chick leg has retained the basic pentadactyl phalangeal pattern in digits 1–4 and therefore is of special interest to the evolution of digit patterns. As we discussed earlier, a model in which Shh signaling specifies different positional values is sufficient to explain chick leg patterning. Thus, any deviations away from this model in the mouse limb would therefore suggest a derived mode of patterning digits 1–4 in the mammalian lineage.

Digit loss has commonly occurred over the course of evolution and alterations in *Shh* expression and response to Shh have been implicated. A striking example is seen in the wings of birds and the fore-limbs of their basal theropod dinosaur ancestors in which two digits have been lost during evolution (Sereno, 1999). Understanding this mode of digit loss has puzzled investigators for over 150 years because theropods appeared to have had digit identities 1, 2, and 3, but in the embryo at least, bird digits appear to arise from positions 2, 3, and 4 (Burke and Feduccia, 1997). Therefore, it was suggested in the so-called “frameshift” model that digits with the identities 1, 2, and 3 arise from positions 2, 3, and 4 of the bird wing (Wagner and Gauthier, 1999; Tamura et al., 2011), perhaps due to reduced Shh signaling levels/duration in limbs of the theropod ancestors of birds (Vargas and Wagner, 2009). However, the Green Fluorescent Protein fate-mapping experiments in chick wings (see Figure 1c) showed that in fact digits with the identities 1, 2, and 3 arise from embryonic positions 1, 2, and 3 that are found in tissue adjacent to the polarizing region (Towers et al., 2011). Therefore, it is not necessary to invoke a frameshift and suggests that the digits 4 and 5 of the dinosaur hand were simply lost and that bird wing digits should be numbered 1, 2, and 3 in line with the fossil record, as is now generally accepted. As already mentioned, in the chick wing bud, the posterior necrotic zone overlaps with the polarizing region. In the chick wing bud, the posterior necrotic zone is much larger than the corresponding zone in chick leg and mouse limb buds (Fernandez-Teran et al., 2006). Therefore, the loss of the two posterior digits in birds might be based on evolutionary changes in Shh signaling, in particular the autoregulatory mechanisms by which Shh signaling regulates apoptosis in the posterior necrotic zone of the wing bud (Sanz-Ezquerro and Tickle, 2000) and also proliferation (Chinnaiya et al., 2014). Interestingly, a recent study showed that an extension of the posterior part of the apical ectodermal ridge in the absence of Shh signaling was sufficient to enable the polarizing region to give rise to a digit in the chick wing. In such buds, the posterior necrotic zone was lost and this was accompanied by a dramatic increase in proliferation of polarizing region cells (Pickering and Towers, 2016).

Shh has also been implicated in digit loss in cow limbs in which only two digits form (3 and 4). It was revealed that *Ptch1* is expressed in the very posterior of the bud and at low levels in response to Shh signaling, because of the degeneration of a cis-regulatory enhancer. As a consequence, it is suggested that Shh fails to be sequestered and restricted to the posterior part of the cow limb bud resulting in more-or-less uniform Shh signaling which results in symmetrical and distally restricted antero-posterior gene expression patterns (Lopez-Rios et al., 2014). As a result, the two digits of the cow limb are also symmetrical and lateral digits are lost because the apical ectodermal ridge fails to extend sufficiently to support their outgrowth. Similarly, *Ptch1* is also restricted to the posterior of the limb buds of pigs that develop four digits, two of which are prominent (digits 3 and 4; Cooper et al., 2014). However, camels do not display a posterior restriction and down-regulation of *Ptch1* in their developing limb buds although they also produce two digits (3 and 4), suggesting another mechanism of digit loss (Cooper et al., 2014). An additional case of digit loss involves the limbs of different species of the Australian skink, *Hemiergis* (Shapiro, 2002). The shortened duration of *Shh* expression in these lizards correlates well with the extent of digit reduction—species with five digits express higher levels of *Shh* for a longer time than those with only two digits (Shapiro et al., 2003). Interestingly, digit reduction correlates with a reduction in cell proliferation. One possibility is that factors other than reduced Shh signaling could be involved. As yet no mutations have been reported in ZRS sequences of various *Hemiergis* clades. However, as further studies are required to understand how positional values are specified by Shh signaling in mammals and lizards, this means that it is difficult to interpret some of the patterns of evolutionary digit loss discussed in this section.

## Future directions

It is now established that Shh has a pivotal function in vertebrate limb development and many details have been uncovered. Surprisingly however, there is still no consensus about how Shh specifies antero-posterior positional values in the limb. It remains possible that different combinations of transcription factors govern antero-posterior positional values, but it has been difficult to identify them because all the digits are made up of the same differentiated cell types. Therefore, a gene-regulatory network such as one operating downstream of Shh in the neural tube to specify distinct neural fates is unlikely to operate during limb development (Balaskas et al., 2012). It is also likely that the temporal regulation of the same sets of genes could contribute to specifying positional values. For instance, there is a clear relationship between *Hoxd* expression and thumb (digit 1) development, with cells that give rise to thumb the only cells that express *Hoxd13* and not *Hoxd12* (Vargas and Fallon, 2005). Therefore, since the cells that give rise to all the other digits express *Hoxd12* and *Hoxd13*, a simple *Hox* code is unlikely to specify the digits, and perhaps timing of expression is the important determinant. Another challenge is to understand how the positional information conferred by Shh signaling is remembered and then interpreted so that digits with different identities arise in the proper places in the limb. In chick limbs, it is clear that the concentration/duration of Shh is sufficient to specify digit identity, however, this is not readily apparent in mammalian limbs because the digits are morphologically similar—at least in terms of phalangeal number. It will be important to fill this gap in knowledge in order to apply the principles to developing human limbs and gain deeper insights into the basis of congenital limb defects and to evolutionary alteration in digit pattern. The analysis of the function of Shh in new animal models of limb development could help resolve issues regarding the relationship between positional values and digit identity. Further development of the CRISPR/Cas9 system should facilitate this.

An issue of general relevance is the mode of Shh transport in the limb and how a graded distribution of Shh is established. This may require further refinement of *in vivo* imaging techniques to visualize directly the distribution of Shh in real time. It also seems clear that the timing of *Shh* expression is another critical parameter that still needs to be addressed. Disentangling the relationship between autoregulatory mechanisms of intrinsic timing of Shh expression and extrinsic mechanisms could shed light on processes that ensure robustness of limb development and pattern scaling between different species.

## Author contributions

MT and CT jointly wrote the manuscript, MT formatted the Figures.

### Conflict of interest statement

The authors declare that the research was conducted in the absence of any commercial or financial relationships that could be construed as a potential conflict of interest.
